# Consequences of Oxidative Stress on Plant Glycolytic and Respiratory Metabolism

**DOI:** 10.3389/fpls.2019.00166

**Published:** 2019-02-18

**Authors:** Sébastien Dumont, Jean Rivoal

**Affiliations:** Institut de Recherche en Biologie Végétale, Département de Sciences Biologiques, Université de Montréal, Montreal, QC, Canada

**Keywords:** oxidative stress, glycolysis, respiration, TCA cycle, redox post-translational modifications, *S*-glutathionylation, *S*-nitrosylation, plant

## Abstract

Reactive oxygen species (ROS) and reactive nitrogen species (RNS) are present at low and controlled levels under normal conditions. These reactive molecules can increase to high levels under various biotic and abiotic conditions, resulting in perturbation of the cellular redox state that can ultimately lead to oxidative or nitrosative stress. In this review, we analyze the various effects that result from alterations of redox homeostasis on plant glycolytic pathway and tricarboxylic acid (TCA) cycle. Most documented modifications caused by ROS or RNS are due to the presence of redox-sensitive cysteine thiol groups in proteins. Redox modifications include Cys oxidation, disulfide bond formation, *S*-glutathionylation, *S*-nitrosylation, and *S*-sulfhydration. A growing number of proteomic surveys and biochemical studies document the occurrence of ROS- or RNS-mediated modification in enzymes of glycolysis and the TCA cycle. In a few cases, these modifications have been shown to affect enzyme activity, suggesting an operational regulatory mechanism *in vivo*. Further changes induced by oxidative stress conditions include the proposed redox-dependent modifications in the subcellular distribution of a putative redox sensor, NAD-glyceraldehyde-3P dehydrogenase and the micro-compartmentation of cytosolic glycolytic enzymes. Data from the literature indicate that oxidative stress may induce complex changes in metabolite pools in central carbon metabolism. This information is discussed in the context of our understanding of plant metabolic response to oxidative stress.

## Introduction

In this review, we examine the effects of oxidative stress on enzymes from glycolysis and the TCA cycle and discuss its impact on various aspects of plant non-photosynthetic carbon metabolism. In recent years, a number of studies have addressed the impact of oxidative stress conditions on plant metabolism. Several proteomic surveys aimed at identifying different types of protein redox modification have also identified enzymes related to carbon metabolism that are targeted by these modifications (Zaffagnini et al., 2012a; Hu et al., 2015; Aroca et al., 2017a). Redox regulation of chloroplastic enzymes form Calvin–Benson cycle has been the subject of several reviews (Ruelland and Miginiac-Maslow, 1999; Buchanan et al., 2002; Michelet et al., 2013). Redox regulation of starch metabolic enzymes has also been evaluated lately (Skryhan et al., 2018). However, the general impact of redox modifications on enzymes from plant glycolytic and respiratory metabolism and on the activity of these pathways has not been assessed recently. Our review first considers protein redox modifications induced by ROS and RNS, as well as antioxidant strategies developed by plants. We then examine the effects of oxidative modifications on glycolytic TCA cycle enzymes to highlight some recent advances. We also discuss the redox regulation of these pathways while addressing other consequences of oxidative stress on these enzymes such as changes in localization, formation of protein complexes and moonlighting properties.

### Origin of Reactive Oxygen Species, Reactive Nitrogen Species, and Hydrogen Sulfide

Reactive oxygen species and RNS are present in plant cells in normal conditions, mainly as by-products of aerobic and photosynthetic metabolism (Tripathy and Oelmüller, 2012). An elevation in ROS and RNS concentrations resulting from the exposure of plants to several biotic and abiotic stresses is also well documented (Airaki et al., 2012; Marino et al., 2012; Romero-Puertas et al., 2013; del Rio, 2015; Demidchik, 2015; Domingos et al., 2015; You and Chan, 2015; AbdElgawad et al., 2016; Camejo et al., 2016). Oxidative stress is caused by such increase in ROS levels leading to the generation of superoxide anion (O_2_^–•^), H_2_O_2_, and hydroxyl radical (OH^∙^) (Thannickal and Fanburg, 2000; Sies et al., 2017). Nitrosative stress is caused by elevated levels of RNS such as NO, peroxynitrite (ONOO^-^) and nitrosoglutathione (GSNO) (Sies et al., 2017; Umbreen et al., 2018). Imbalance in ROS and RNS concentrations during stress can lead to various types of damage to cell macromolecules, including proteins (Apel and Hirt, 2004; Møller et al., 2007; Foyer and Noctor, 2009; Corpas et al., 2011).

**FIGURE 1:**
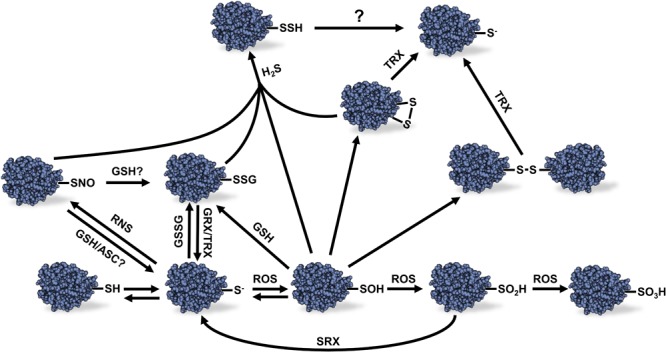
Different redox modifications of protein Cys thiols and their reversibility. Protein thiol oxidation can lead to a variety of redox modifications. The scheme presents the relations between the different redox modifications and the reversibility of these modifications. Sulfonic acid (–SO_3_H) formation is considered to be irreversible as well as sulfinic acid (–SO_2_H) except in the case of certain peroxiredoxins which sulfinic acid Cys residue can be reduced by sulfiredoxin using and ATP-dependent mechanism. See text for details. ROS, reactive oxygen species; RNS, reactive nitrogen species; H_2_S, hydrogen sulfide; GSSG, oxidized glutathione; GSH, reduced glutathione; ASC, ascorbate; GRX, glutaredoxin; TRX, thioredoxin; SRX, sulfiredoxin; R-SH, reduced thiol; R-S^-^, thiolate anion; R-SOH, sulfenic acid; R-SNO, protein *S-*nitrosylation; R-SSG, protein *S*-glutathionylation Cys; R-SSH, protein *S*-sulfhydration; R-SS-R, disulfide bond.

In plants, ROS can be generated by the ETC in chloroplasts and mitochondria. Membrane-localized NADPH oxidases are also known as ROS producers during plant development and in response to stress (Marino et al., 2012). GSH is known to be involved in redox buffering (see below), but is also known for its ability to form disulfide bridges with sensitive protein Cys residues resulting in a redox PTM named *S*-glutathionylation (Figure 1) (Zaffagnini et al., 2012c). NO can be produced in mitochondria through the reduction of NO_2_^–^ by the mitochondrial ETC and possibly via nitrite and arginine dependent pathways (Gupta and Kaiser, 2010; Igamberdiev et al., 2010; Zaffagnini et al., 2016). Nitrosoglutathione (GSNO)/GSNO reductase system, peroxiredoxins, TRXs, and pathogens- and hypoxically induced non-symbiotic hemoglobin are known to participate in the control of NO (Dordas et al., 2003a,b, 2004; Seregélyes et al., 2003; Bailey-Serres and Voesenek, 2008; Gupta and Igamberdiev, 2011; Zaffagnini et al., 2016).

Hydrogen sulfide is a gaseous signaling molecule found in both plants and animals. In plants, H_2_S is produced by the sulfur assimilation pathway and L-/D-cysteine degradation (Fu et al., 2018). High levels of H_2_S can be phytotoxic, but at lower concentrations, H_2_S was demonstrated to play several roles during plant growth and development as well as biotic and abiotic stress response (Zhang et al., 2008; Chen et al., 2011; Shen et al., 2012, 2013). As opposed to ROS and RNS, H_2_S is not an electrophilic molecule that spontaneously reacts with reduced thiol groups. However, H_2_S can react with oxidized thiols leading to protein *S*-sulfhydration (also named persulfidation) (Figure 1) (Paul and Snyder, 2012).

### Antioxidant Strategies in Plants

In order to cope with oxidative stress, plants have developed several enzymatic and non-enzymatic strategies to scavenge free ROS in cells. Today, these mechanisms are well known and have been extensively reviewed (Mittler, 2002; Gill and Tuteja, 2010; Sharma et al., 2012). Non-enzymatic strategies depend on small molecules such as membrane localized α-tocopherols which can play essential antioxidant functions by acting as “sacrificial chemical scavengers” (Fryer, 1992), leading to the removal of ROS molecules through oxidation of α-tocopherols. Plastid localized carotenoids also protect plant cells from oxidative stress similarly to α-tocopherols (Larson, 1988; Mittler, 2002). The amino acid Pro is known to be induced by oxidative stress and has been shown to have a positive effect on antioxidant enzymes in plants (Ozturk and Demir, 2002). It has also been demonstrated that Pro can scavenge hydroxyl radicals, suggesting an important role of this amino acid in tolerance to oxidative stress (Smirnoff and Cumbes, 1989; Gill and Tuteja, 2010). Plants also accumulate other antioxidant molecules such as flavonoids (Hernandez et al., 2009) and polyamines (Groppa et al., 2001) that were shown to increase tolerance to several environmental stresses. Small redox active molecules such as ASC and the Cys-containing tripeptide GSH can also be used with specific peroxidase systems for ROS removal. Once oxidized, ASC and GSH can be recycled to their reduced form by reductases systems using NADPH as reductive power (Noctor et al., 2017).

Antioxidant enzymes and enzymatic-dependent mechanisms also control ROS levels in plant cells. Superoxide dismutase is a metalloenzyme responsible for the detoxification of superoxide anion leading to the formation of H_2_O_2_ and O_2_ (Alscher et al., 2002). H_2_O_2_ can be metabolized by several antioxidant enzymes such as catalase, ASC peroxidase (APX), glutathione peroxidase (GPX) and peroxiredoxins (PRXs). As opposed to different kind of peroxidases, catalase does not require reductant to detoxify H_2_O_2_ (Mhamdi et al., 2010). The H_2_O_2_ metabolizing enzyme APX catalyzes the first step of the ASC-GSH cycle. In this process, ASC is oxidized to dehydroascorbate (DHA) by APX to detoxify H_2_O_2_. DHA is then recycled by a DHA reductase using GSH as reducing power, thus producing GSSG. The latter is reduced back to GSH by GSH reductase using NADPH as a reductant (Noctor and Foyer, 1998). Similarly, to APX, GPX can detoxify H_2_O_2_ but using directly GSH instead of ASC as reducing power (Margis et al., 2008). Like APX and GPX, PRXs metabolize organic hydroperoxides such as H_2_O_2_. PRXs use Cys residues in their protein sequence as reducing power for their enzymatic activity. Briefly, PRXs react with H_2_O_2_ via a catalytic Cys residue which becomes oxidized leading to the formation of inter- or intra-molecular disulfide bond depending on their type (Dietz, 2003). Some PRXs can also form a disulfide bond with glutathione (Noguera-Mazon et al., 2006). Once oxidized under disulfide form, PRXs are reduced back by a disulfide reductase system such as TRXs or GRXs.

### Cys Oxidation by ROS Molecules

Despite the presence of antioxidant systems in plant cells, ROS can increase to potentially harmful levels in specific conditions leading to oxidative stress. Protein Cys residues are major targets of redox modifications due to the chemical properties of the sulfur atom present in thiol groups (Figure 1). Protonated thiol groups display a lower sensitivity to oxidation by electrophilic molecules like ROS than the deprotonated thiolate anion form. Usually, Cys thiols tend to stay mostly protonated in physiological conditions due to their p*K*_a_ around 8.5–9.0. However, the p*K*_a_ of some Cys residues can be lowered when the thiol group is sterically near the side chain of basic amino acids such as Arg, Lys, or His; as well as in the proximity of metal ions, alpha helices dipole or specific H-bonding in the 3D structure of the protein (Britto et al., 2002; Barford, 2004; Zaffagnini et al., 2012c). These conditions will promote deprotonation of thiol groups and stabilize the thiolate anion form making these Cys residues more nucleophilic, therefore more reactive toward ROS (Finkel, 2000; Choi et al., 2001). Cys oxidation by ROS leads to the formation of a sulfenic acid. This latter group is highly unstable and can be further oxidized to sulfinic acid (R-SO_2_H) and sulfonic acid (R-SO_3_H) (Figure 1). In most cases, these latter modifications are considered to be non-reversible *in vivo* (Ghezzi and Bonetto, 2003; Forman et al., 2010) except for some reported PRXs for which Cys sulfinic acid could be reduced by a sulfiredoxin using an ATP-dependent mechanism (Chang et al., 2004; Woo et al., 2005). Sulfenic acid can also react with a reduced thiol group to form intra- or inter-protein disulfide bonds, or a disulfide bond with GSH leading to protein *S*-glutathionylation (Figure 1). These redox PTMs can be reversed *in vitro* by addition of reductants such as dithiothreitol (DTT) or *in vivo* by specific oxidoreductases such as TRXs or GRXs (Poole et al., 2004; Zaffagnini et al., 2012c).

### Protein *S*-Glutathionylation, *S*-Nitrosylation, and *S*-Sulfhydration

Protein *S-*glutathionylation can prevent unwanted disulfide bonds, aggregates and irreversible Cys oxidation in cells by promoting spontaneous disulfide bond formation between protein sulfenic acid Cys and glutathione (Rouhier et al., 2008; Zaffagnini et al., 2012c). Protein *S*-glutathionylation can also occur spontaneously by reaction of a reduced Cys residue with GSSG (Figure 1). However, due to the high GSH:GSSG ratio in the cytosol and mitochondria, *S*-glutathionylation by thiol disulfide exchange in these compartments seems unlikely to happen *in vivo* unless during exposure to severe oxidative stress conditions (Meyer et al., 2007; Schwarzländer et al., 2008). Interestingly, addition of GRXC2 was shown to accelerate *A. thaliana* BRI1-associated receptor-like kinase 1 inhibition by GSSG *in vitro* suggesting a first, and still unique, example of *S*-glutathionylation catalysis in plants (Bender et al., 2015). The reverse reaction of protein *S*-glutathionylation is called deglutathionylation (Figure 1). While some TRXs were shown to be able to perform deglutathionylation reactions *in vitro*, their efficiency to catalyze this reaction is poor compared to GRXs (Bedhomme et al., 2012). It should be noted, however, that the involvement of GRXs and TRXs in deglutathionylation depends on conditions prevailing *in vivo*, including expression level of the two types of proteins. GRXs are small proteins with GSH-dependent disulfide oxidoreductase activity present in almost all living organism. GRXs belong to the TRX superfamily and can be categorized in three different classes, depending on the structure of their active site. GRXs have been well studied and reviewed in photosynthetic organisms (Rouhier et al., 2008; Zaffagnini et al., 2012b,c).

Like ROS, RNS such as NO can react with redox sensitive Cys thiols to form nitrosothiols (-SNO) in the process of protein *S*-nitrosylation (Wilson et al., 2008) (Figure 1). This modification occurs via the direct reaction of a Cys thiolate with RNS molecules, metal-mediated nitrosylation or trans-nitrosylation by *S-*nitrosothiols such as GSNO (Zaffagnini et al., 2016). This latter molecule results from the *S*-nitrosylation of cellular GSH. The mechanism of GSNO formation is not yet fully understood, but likely involves the *S*-nitrosylation of GSH by NO. GSNO is known as an important NO reservoir and donor in plant cells and is also able to induce protein *S*-glutathionylation (Zaffagnini et al., 2016). Furthermore, it is known that *S-*nitrosylated protein thiols can become *S*-glutathionylated by GSH (Mohr et al., 1999). The conditions that lead to *S*-nitrosylation or *S-*glutathionylation by GSNO still remain unclear. The reverse reaction of protein *S*-nitrosylation, denitrosylation, can be favored by reductant such as ASC, DTT and GSH. *In vivo* denitrosylation is though to be related to TRX and GSNO reductase systems (Benhar et al., 2009).

Protein *S*-sulfhydration is an emerging redox modification promoted by H_2_S that can modify Cys residues on protein to form persulfide groups (R-SSH) as mentioned above (Figure 1). The molecular mechanism leading to protein *S*-sulfhydration remains unclear. It was proposed that H_2_S could react with an oxidized Cys residue or with ROS to form a more reactive intermediate which could react with sensitive thiolate anions (Finkel, 2012; Sen et al., 2012; Couturier et al., 2013). Little is known about the effects of protein *S*-sulfhydration in plants. In animals, *S*-sulfhydration of glyceraldehyde-3-phosphate dehydrogenase (GAPDH) was originally shown to increase its enzyme activity (Mustafa et al., 2009) as opposed to *S*-glutathionylation and *S*-nitrosylation that are known to inactivate the enzyme (Padgett and Whorton, 1995; Cotgreave et al., 2002). However, the effect of *S*-sulfhydration on GAPDH was later revisited and the modification of Cys156 was shown to inhibit enzyme activity (Jarosz et al., 2015). The sensitivity of animal GAPDH activity to different redox modifications nevertheless suggests complex interactions between various redox signals.

## Effects of Redox Modifications of Glycolytic, Fermentative and Respiratory Metabolism Enzymes

### Glycolysis and Fermentation

**FIGURE 2:**
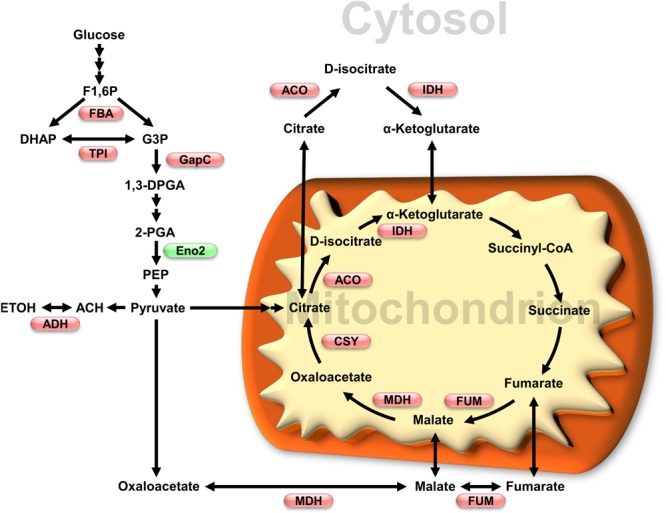
Impact of oxidative stress on glycolytic and TCA cycles enzymes activity. Enzymes which activity has been shown to be affected by redox modification are shown in a metabolic scheme that also includes subcellular localization. Red and green, respectively, indicate inhibition and activation under oxidative conditions. Enzymes and metabolites abbreviations: FBA, fructose-1,6-bisphosphate aldolase; TPI, triosephosphate isomerase; GapC, glyceraldehyde 3-phosphate dehydrogenase; Eno2, enolase 2; ADH, alcohol dehydrogenase; ACO, aconitase; IDH, isocitrate dehydrogenase; MDH, malate dehydrogenase; FUM, fumarase; CSY, citrate synthase. Metabolites: F1,6P, fructose-1,6-bisphosphate; DHAP, dihydroxyacetone phosphate; G3P, glyceraldehyde 3-phosphate; 1,3DPGA, 1,3-bisphosphoglycerate; 2-PGA, 2-phosphoglycerate; PEP, phospho*enol*pyruvate; ACH, acetaldehyde; ETOH, ethanol.

Redox regulation of sugar-related metabolic pathways seems to have a fundamental role in photosynthetic organisms. For example, many enzymes from starch metabolism were shown to be redox-sensitive and suggested to be regulated in function of the light/dark dependent redox state of chloroplast (Skryhan et al., 2018). In plants, the glycolytic pathway exists in the cytosol and the plastids (Plaxton, 1996). Many chloroplastic isoforms of glycolytic enzymes take part in the Calvin-Benson pathway, for which redox regulation has been well studied (Michelet et al., 2013). However, less information is available on redox regulation of the cytosolic glycolytic pathway in plants, except for NAD-dependent GAPDH which has been used as a model for protein redox PTMs (Holtgrefe et al., 2008; Bedhomme et al., 2012; Zaffagnini et al., 2014; Hildebrandt et al., 2015). Several enzymes from the cytosolic glycolytic pathway have been identified in different surveys as potential targets of redox modifications in *A. thaliana* (Table 1). Nevertheless, few of these identified targets have also been studied to understand the impact of redox modification on their protein function, including enzymatic activity. Here we analyze literature data on cytosolic glycolytic enzymes which activity and functions were shown to be affected by Cys redox modifications (Figure 2).

**Table 1:** List of different redox modifications of *A. thaliana* proteins from glycolysis, fermentation, and TCA cycle identified from literature data.

Cytosolic/mitochondrial enzymes	*S-*glutathionylation	*S-*nitrosylation	*S-*sulfhydration	Other redox modifications or unknown
**GLYCOLYSIS**				
**Suc synthase**				
*SUS4 (At3g43190)*	1	8		
*SUS1 (At5g20830)*	1	8 [Cys^349/731^]		
*Like protein (At5g20280)*			13	
**Phosphoglucomutase**				
*PGM3 (At1g23190)*		8	13	
*PGM2 (At1g70730)*		8	13	
**Glucose 6 phosphate isomerase**				
*PGIC (At5g42740)*			13	
**Phosphofructokinase**				
*PFP- PFK (At1g20950, At1g76550)*		8 [Cys^28/586^]		
*PFP- PFK (At1g12000)*		8 [Cys^166^]		
*PFK7 (At5g56630)*			13	
*PFK1 (At4g29220)*			13	
*PFK3 (At4g26270)*			13	
*PFK6 (At4g32840)*			13	
**Fructose-bisphosphate aldolase**				
*FBA8 (At3g52930)*	1	9	13, 14	19
*FBA6 (At2g36460)*	2 [Cys^68/173^]	2 [Cys^173^], 8	13	
*FBA4 (At5g03690)*		8	13	
*FBA5 (At4g26530)*			13, 14	
*FBA7 (At4g26520)*			13	
**Triosephosphate isomerase**				
*cTPI* (*At3g55440)*	3, 4 [Cys^127/218^]	8 [Cys^127^], 9, 10 [Cys^127/218^], 11 [Cys^127/218^]	13, 14	17 [Cys^13^], 19
**Glyceraldehyde phosphate dehydrogenase**				
*GapC1 (At3g04120)*	1, 5 [Cys^155^], 6	8 [Cys^155^], 9, 12 [Cys^155^]	13, 16, 15 [Cys^160^]	19, 20
*GapC2 (At1g13440)*	1, 6 [Cys^155^]	6 [Cys^155/159^]	13, 14	19, 20
**Phosphoglycerate kinase**				
*PGK3 (At1g79550)*		9	13	19
**Phosphoglycerate mutase**				
*PGAM1 (At1g09780)*		8, 10 [Cys^100^]	13	16
*PGAM like (At5g64460)*		9 [Cys^103^]	13	
*Family protein (At5g62840)*			13	
**Enolase**				
*ENO2 (At2g36530)*	1	8 [Cys^318^], 9	13, 14	19
**Pyruvate kinase**				
*Family protein (At5g56350)*		8 [Cys^113/154/425^]	13	16
*PK (At3g52990; At2g36580; At5g08570; At3g55650; At4g26390)*			13	
*PK (At5g63680)*			13	16
**PEPC**				
*PEPC1 (At1g53310)*		8 [Cys^420^]	13	
*PPC2 (At2g42600)*		8 [Cys^419^]	13	
**FERMENTATION**				
**Lactate dehydrogenase**				
*LDH (At4g17260)*			13	
**Pyruvate decarboxylase**				
*PDC (At5g54960)*	1		13	17 [Cys^554^]
*PDC (At4g33070)*	1		13	
**Alcohol dehydrogenase 1**				
*ADH (At1g77120)*	1, 7 [Cys^47/243^]	7, 10 [Cys^243^]	13	19
**TCA CYCLE**				
**Citrate synthase**				
*CSY4 (At2g44350)*		8 [Cys^326/468^]	13	21 [Cys^365^]
**Aconitase**				
*ACO1 (At4g35830)*		8 [Cys^396^]	13, 14	16
*ACO2 (At4g26970)*		8 [Cys^493^]	13	16
*ACO3 (At2g05710)*		8 [Cys^488^], 9	13	17 [Cys^961^]
**Isocitrate dehydrogenase**				
*IDH1 (At4g35260)*		8	13	22 [^C128/216^]
*IDH2 (At2g17130)*		8	13	
*NADP-IDH (At1g65930)*		10 [Cys^75/262/363^], 11 [Cys^75/363^]	13	17 [Cys^116^], 18 [Cys^75/116/131/269^]
**Succinyl-CoA ligase**				
*SCS (At2g20420, At5g23250)*			13	
*SCS (At5g08300)*			13	16
**Succinate dehydrogenase**				
*SDH1 (At5g66760)*		10 [Cys^526^]	13	
*SDH5 (At1g47420)*				17 [Cys^230^]
**Fumarase**				
*FUM1 (At2g47510)*	1	8 [Cys^155^]	13	17 [Cys^155^], 23
*FUM2 (At5g50950)*				23 [Cys^339^]
**Malate dehydrogenase**				
*cyto-MDH1 (At1g04410)*		9	13, 14	18 [Cys^155^], 24 [Cys^330^], 25 [Cys^330^]
*cyto-MDH2 (At5g43330)*		8	13	19
*mito-MDH2 (At3g15020)*		9	13	
*mito-MDH1 (At1g53240)*				19, 20, 26

*Numbers in columns refer to the publications in the footnotes. When identified, modified residues are indicated between brackets.*

*1, (Dixon et al., 2005); 2, (van der Linde et al., 2011); 3, (Ito et al., 2003); 4, (Dumont et al., 2016); 5, (Bedhomme et al., 2012); 6, (Holtgrefe et al., 2008); 7, (Dumont et al., 2018); 8, (Hu et al., 2015); 9, (Lindermayr et al., 2005); 10 (Fares et al., 2011); 11, (Puyaubert et al., 2014); 12, (Zaffagnini et al., 2013b); 13, (Aroca et al., 2017a); 14, (Aroca et al., 2015); 15, (Aroca et al., 2017b); 16, (Akter et al., 2015); 17, (Liu et al., 2014); 18, (Alvarez et al., 2009b); 19, (Wang et al., 2012); 20, (Alvarez et al., 2009a); 21, (Schmidtmann et al., 2014); 22, (Yoshida and Hisabori, 2014); 23, (Zubimendi et al., 2018); 24, (Hara et al., 2006); 25, (Huang et al., 2018); 26, (Yoshida and Hisabori, 2016).*

Cytosolic FBA catalyzes the reversible interconversion of fructose-1,6-bisP to glyceraldehyde 3P and dihydroxyacetone phosphate. FBA6 from *A. thaliana* was shown to undergo *S-*glutathionylation *in vitro* (van der Linde et al., 2011). Incubation of recombinant FBA6 in the presence of GSSG or GSNO led to FBA6 activity inhibition by *S*-glutathionylation. The presence of fructose-1,6-bisphosphate, protected it from inhibition by both treatments. ESI-MS analysis of FBA6 identified *S*-glutathionylation of Cys68 after incubation with GSSG. Interestingly, ESI-MS analysis showed *S-*nitrosylation and *S-*glutathionylation of Cys173 after treatment with GSNO, suggesting that the two modifications were induced by GSNO. FBA6 was also shown to co-localize with TRX*h* in the cytosol and in nucleus using *A. thaliana* protoplasts (van der Linde et al., 2011). However, it is not clear if FBA6 subcellular localization and co-localization with TRX*h* is related to redox modification of the enzyme.

Cytosolic triosephosphate isomerase equilibrates the pools of fructose-1,6-bisP and glyceraldehyde 3P and dihydroxyacetone phosphate. *S*-glutathionylation of cTPI was originally detected *in vivo* in *A. thaliana* cell cultures treated with BioGEE, a biotinylated analog of GSH (Ito et al., 2003). Recombinant cTPI was shown to be sensitive to inhibition by treatments inducing proteins *S*-glutathionylation such as GSSG and diamide + GSH (Ito et al., 2003; Dumont et al., 2016). Inactivation of cTPI by *S*-glutathionylation could be reversed enzymatically by cytosolic GRXC1 and GRXC2 (Dumont et al., 2016). Moreover, two Cys residues (Cys127 and Cys218) were identified as potential target of *S*-glutathionylation. Mutation of Cys127 to Ser caused a large decrease in the enzyme activity while mutation of Cys218 to Ser had less effect on cTPI activity. Inhibition of the Cys218 mutant by *S*-glutathionylation was similar to the inhibition measured for the wild type cTPI, meaning that redox modification of this residue was not responsible for the loss of cTPI activity. These results suggest that Cys127 is probably the major target of *S*-glutathionylation and is more important for enzyme activity (Dumont et al., 2016). Both Cys127 and Cys218 residues were also found as potential *S*-nitrosylation targets in *A. thaliana* (Fares et al., 2011; Hu et al., 2015). cTPI was also found as a putative target of other redox modifications (Table 1). It is reasonable to hypothesize that *S*-glutathionylation of cTPI occurring *in vivo* would result in metabolic effects similar to those found in cTPI antisense roots, where a decrease in cTPI was shown to result in a partial redirection of the glycolytic flux to the PPP (Dorion et al., 2012; Valancin et al., 2013).

NAD-dependent GAPDH catalyzes the oxidation and phosphorylation of glyceraldehyde 3P in the cytosol to produce 1,3-bisphosphoglycerate and NADH. This enzyme is well known to undergo several redox PTMs and to display moonlighting functions in plant cells (Zaffagnini et al., 2014; Hildebrandt et al., 2015). For these reasons, cytosolic NAD-dependent GAPDH (GapC) is considered as a redox sensor in plant cells (Morigasaki et al., 2008; Hildebrandt et al., 2015; Schneider et al., 2018). The two cytosolic isoforms of GapC (GapC1 and GapC2) undergo *S*-glutathionylation *in vivo* after incubation of *A. thaliana* cell cultures with biotinylated GSSG (BioGSSG) (Dixon et al., 2005). Recombinant *A. thaliana* GapC1 and GapC2 showed a decrease in their activity after incubation with GSNO, H_2_O_2_, GSH + H_2_O_2_ and GSSG (Holtgrefe et al., 2008; Bedhomme et al., 2012). After treatment with GSH + H_2_O_2_ or GSSG, GapC1 activity could be recovered by addition of DTT, however, inhibition caused by H_2_O_2_ alone led to an irreversible modification (Bedhomme et al., 2012). Site directed mutagenesis demonstrated that Cys155 is essential for GapC1 activity while mutation of Cys159 did not affect enzyme activity. While both Cys residues are involved in substrate binding, only the highly acidic Cys155 along with His181 have catalytic functions in the active site (Zaffagnini et al., 2013a). Deglutathionylation of GapC1 could be performed *in vitro* by different isoforms of TRXs as well as GRXC1, the latter being significantly more efficient (Bedhomme et al., 2012). Recombinant GapC1 was also shown to undergo *S*-nitrosylation on Cys155, leading to inhibition of the enzyme (Zaffagnini et al., 2013b). Inhibition was reversible with addition of GSH or DTT. However, TRXs were ineffective for recovering GapC1 activity from the nitrosylated enzyme. Redox-dependent inhibition of GapC1 and GapC2 activities would have direct consequences on plant primary metabolism. Indeed, *A. thaliana* double KO mutants for both GapC isoforms showed reduced levels of ATP, downstream glycolytic intermediates and organic acids from the TCA cycle (Guo et al., 2014). These results suggest a lower glycolytic flux to pyruvate due to the lack of GapCs. The double KO mutants also had a higher NADPH/NADP ratio. It is thus possible that low GapCs activity could increase carbon flux through PPP similarly to what was proposed for low cTPI (Dorion et al., 2012; Valancin et al., 2013; Guo et al., 2014). KO lines showed lower oil accumulation in seeds probably due to the reduction in precursors and energy required for fatty acid synthesis, thus altering lipid metabolism (Guo et al., 2014).

The GapC1 isoform, has been shown to accumulate in the cell nucleus during abiotic or biotic stresses (Vescovi et al., 2013; Henry et al., 2015; Testard et al., 2016) suggesting a moonlighting function in response to oxidative stress. Cd stress was shown to increase NO in *A. thaliana* root tips. Accumulation of GapC1 in cell nucleus was demonstrated after Cd treatment and also after treatment with L-BSO, an inhibitor of GSH biosynthesis which is known to induce oxidative stress in plants (Vescovi et al., 2013). Interestingly, mutation of Cys155 to Ser did not prevent GapC1 accumulation in the nucleus, suggesting that redox modification of this Cys during Cd stress is not required for its translocation to the nucleus (Vescovi et al., 2013). Accumulation of GapC in cell nucleus was also shown following stress induced by H_2_O_2_ or GSNO in *A. thaliana* protoplasts (Schneider et al., 2018). In this case, mutation of both Cys156 and Cys160 prevented an increase of GapC localization in cell nucleus following oxidative stress treatments. These results suggest that redox modification of GapC Cys residues is necessary for its higher nucleus accumulation under stress caused by H_2_O_2_ or GSNO (Schneider et al., 2018). The difference in localization following stress with the previous study (Vescovi et al., 2013) could be related to the identity of the stressing agent. Accumulation of GapC in the nucleus was also observed in tobacco BY-2 cells treated with DHS (Testard et al., 2016). DHS is known to trigger an apoptotic-like response in tobacco, and in parallel, to increase ROS and RNS production (Lachaud et al., 2010, 2011; Da Silva et al., 2011). GapC1 accumulation in the nucleus was found to be dependent on NO levels in tobacco cells. Indeed, addition of the NO scavenger [2-(4-carboxyphenyl)-4,4,5,5-tetramethylimidazoline-1-oxyl 3-oxide] (cPTIO) prevented accumulation of GapC1 in the nucleus in DHS-treated tobacco cells while addition of a NO donor showed increase in nuclear localization (Testard et al., 2016). A biotin-switch assay also demonstrated that the GapC enzyme present in the nucleus was *S*-nitrosylated. Expression of GapC1 and GapC2 carrying a double mutation for Cys155 and Cys159 in tobacco showed localization to the nucleus (Testard et al., 2016). This supports previous results obtained in Cd stressed *A. thaliana* root tips in which the redox modification of GapC1 and GapC2 is not a prerequisite to the migration of these enzymes to the nucleus (Vescovi et al., 2013). These data suggest a different mechanism, as proposed in animals, where *S*-nitrosylation of GAPDH promotes binding with Siah1 followed by translocation of the Siah1–NO–GAPDH complex into the nucleus (Hara et al., 2005). Interestingly, GapC1 and GapC2 were both able to bind nucleic acids in tobacco while their mutated forms were not (Testard et al., 2016). GapC1 and GapC2 were also identified as putative targets of *S*-sulfhydration in *A. thaliana* (Aroca et al., 2015, 2017a). LC-MS/MS analysis showed modification of Cys159 by *S*-sulfhydration and treatment with NaHS of *A. thaliana* plants was proposed to increase localization of GapC in the nucleus (Aroca et al., 2017b). GapC was also demonstrated to interact with the plasma membrane–associated PLDδ under oxidizing conditions. Indeed, H_2_O_2_ was shown to promote binding of GapC1 and GapC2 to PLDδ. Interaction between GapC and PLDδ was proposed to increase phosphatidic acid by PLDδ, which could stimulate ROS production via NADPH oxidase. Interaction of oxidized GapC with PLDδ was shown to play important functions for growth inhibition response to drought stress (Guo et al., 2012).

Enolase converts 2-phosphoglycerate to phospho*enol*pyruvate (PEP), the immediate precursor of pyruvate in the glycolytic pathway and a branch point to the shikimic acid pathway. Three *ENO* genes are present in *Arabidopsis*. *ENO1* encodes a weakly expressed plastidic isoform, while *ENO2* and *ENO3* encode cytosolic isoforms (Troncoso-Ponce et al., 2018). ENO2 is the only active cytosolic ENO isoform (Andriotis et al., 2010). It was detected as a putative target of several redox modifications (Table 1). ENO2 is thought to be the main ENO isoform operating in most plant cells. Little is known about the effect of these modifications on the enzyme activity, however, addition of diamide in *A. thaliana* and ice plant cell extracts was reported to increase ENO activity (Anderson et al., 1998). Diamide is a thiol-specific oxidant that promotes protein *S*-glutathionylation *in vivo* and *in vitro* (Michelet et al., 2008; Dumont et al., 2016). The authors suggested that the increase in ENO activity could be caused by a disulfide bond between Cys318 and Cys346.

Alcohol dehydrogenase catalyzes the reduction of acetaldehyde to ethanol and is the final step of the ethanolic fermentation pathway. It is encoded by a single gene in *A. thaliana* (Strommer, 2011) and is essential for survival in hypoxia (Ismond et al., 2003). Recently, we showed that ADH activity inhibition by H_2_O_2_ treatment in *A. thaliana* cell cultures while oxidative treatment with diamide did not affect the enzyme activity. This suggests an H_2_O_2_-mediated redox modification of this enzyme. While *in vitro S*-glutathionylation of the recombinant enzyme led to the identification of Cys47 and Cys243 as redox sensitive thiols, it did not affect the ADH activity. ADH was, however, sensitive to irreversible inhibition *in vitro* by H_2_O_2_ and reversible inhibition by the NO donor, diethylamine NONOate. Since NAD^+^ or NADH prevented this inhibition at concentrations compatible with those occurring in plant cells, it was hypothesized that nicotinamide adenine dinucleotides could mediate ADH protection *in vivo* (Dumont et al., 2018).

### TCA Cycle and Glyoxylate Cycle

Many TCA cycle enzymes were shown to respond to oxidative stress in plants. Treatment of *A. thaliana* cell cultures with H_2_O_2_ caused the reduction in protein abundance for ACO, the E2 subunit of the pyruvate dehydrogenase complex, 2-oxoglutarate dehydrogenase, FUM, and succinyl CoA ligase (Sweetlove et al., 2002). ACO, IDH, FUM, and MDH were reported as *S*-glutathionylation targets in *C. reinhardtii* (Zaffagnini et al., 2012a). Several proteomic surveys also reported redox modifications of TCA cycle enzymes in *A. thaliana* (Table 1). In spite of these studies, little information is yet available on the possible molecular mechanisms involved in the redox regulation of these enzymes.

Mitochondrial citrate synthase catalyzes the first enzymatic reaction of the TCA cycle by condensing acetyl-CoA and oxaloacetate to form citrate. *A. thaliana* also possesses peroxisome-localized citrate synthase isoforms, which take part in the glyoxylate cycle and fatty acid respiration (Pracharoenwattana et al., 2005). Addition of diamide in crude extract from pummelo fruit, *Solanum tuberosum* and *A. thaliana* decreased citrate synthase activity. Addition of DTT caused an increase in citrate synthase activity in pummelo fruit and *S. tuberosum* crude extracts, however, no significant changes were observed in *A. thaliana* (Stevens et al., 1997). The main mitochondrial citrate synthase isoform (CS4) was shown to be inhibited by H_2_O_2_ (Schmidtmann et al., 2014). The enzyme was also shown to form an aggregate in a redox-dependent manner. Addition of *E. coli* TRX (with NADPH-TRX reductase) resulted in an increase the activity of the enzyme previously purified under non-reducing condition. Interestingly, site directed mutagenesis showed that mutation of Cys365 to Ser reduced the sensitivity of citrate synthase to oxidation and also reduced activation by TRX (Schmidtmann et al., 2014). This Cys residue was also found *S*-nitrosylated in the same isoform of citrate synthase (Hu et al., 2015).

Aconitase catalyzes the reversible isomerisation of citrate to isocitrate in the TCA cycle. ACO is an iron–sulfur containing protein with isoforms localized in mitochondria and cytosol. In the latter case, it participates to the glyoxylate cycle (Eprintsev et al., 2015). ACO was shown to be inactivated by H_2_O_2_ in *S. tuberosum* (Verniquet et al., 1991) and by both H_2_O_2_ and NO in tobacco leaves (Navarre et al., 2000). Following these results, it was proposed that ACO inhibition by ROS or RNS could take part in plant pathogen response. Indeed, redox-dependent inhibition of ACO is suggested to lead to elevated levels of citrate (Navarre et al., 2000; Gupta et al., 2012), which could in turn induce alternative oxidase (Vanlerberghe and Mclntosh, 1996). This process is known to be implicated in resistance to Tobacco Mosaic Virus and other viruses (Chivasa and Carr, 1998; Murphy et al., 1999; Navarre et al., 2000). Induction of alternative oxidase via citrate accumulation due to ACO *S*-nitrosylation during hypoxia was also proposed as a response mechanism to control ROS production in mitochondria during stress conditions (Gupta et al., 2012). Accumulation of citrate due to ACO inhibition during hypoxia was also proposed to cause a metabolic shift toward amino acid biosynthesis as an adaptation strategy (Gupta et al., 2012). Resistance to paraquat treatment was enhanced in ACO-silenced tobacco or *A. thaliana* KO lines, suggesting that inhibition of ACO activity during oxidative stress would help plant to cope with oxidative stress (Moeder et al., 2007). Cytosolic ACO was also found to be related to cell death. This case, it was suggested that Fe^2+^ released by the oxidized enzyme could react with H_2_O_2_ to form hydroxyl radicals that would enhance cell death (Moeder et al., 2007).

Mitochondrial NAD^+^-dependent IDH (mIDH) catalyzes the oxidative decarboxylation of isocitrate and forms 2-oxoglutarate, CO_2_ and NADH. In yeast, IDH is a heterodimer composed of two subunits called IDH-r (regulatory) and IDH-c (catalytic). Yeast IDH is activated by AMP and is inactivated by formation of intermolecular disulfide bonds located on IDH-c subunits (Taylor et al., 2008; Garcia et al., 2009; Yoshida and Hisabori, 2014). In *A. thaliana*, mIDH has a heterodimeric structure formed by IDH-c and IDH-r that is insensitive to adenylates (Yoshida and Hisabori, 2014). Each of these subunits type alone has been shown to be inactive. It was demonstrated that treatment with 50 μM CuCl_2_ induced oxidation of mIDH-r subunit but not mIDH-c. Oxidative inactivation of the enzyme activity could be partially reversed by DTT. Non-reducing SDS-PAGE analysis showed the formation of IDH-r trimers following oxidation of the protein. Oxidized IDH-r trimers could be reduced back to monomers on non-reducing gels with high concentrations of DTT. Lower DTT concentrations were needed to reduce oxidized IDH-r in presence of the mitochondrial TRX*o1* (Yoshida and Hisabori, 2014), suggesting redox regulation of this enzyme by TRXs as proposed before (Yoshida et al., 2013). Mass spectrometry analysis identified that Cys128 and Cys216 were involved in the formation of disulfide bonds in IDH-r.

Cytosolic NADPH-IDH activity measured from *A. thaliana* leaves and roots using the assumption that the cytosolic isoform corresponds to 90% of the total activity (Galvez and Gadal, 1995; Chen, 1998). Extracts were treated with H_2_O_2_, 3-morpholinosydnonimine (SIN-1, a NO donor) and GSNO. H_2_O_2_ decreased NADPH-IDH activity in roots but not in leaves. Both SIN-1 and GSNO had inhibitory effects on IDH activity in roots and leaves. Interestingly, addition of GSH in leaves extracts also decreased IDH activity (Leterrier et al., 2012).

Fumarase catalyzes the reversible conversion of fumarate to L-malate. *A. thaliana* possesses two genes for FUM isoforms. FUM1 is localized in mitochondria where it plays an essential role in TCA cycle. FUM2 is cytosolic and is required for accumulation of fumarate during the day (Pracharoenwattana et al., 2010). Accumulation of fumaric acid due to cytosolic FUM2 activity was shown to be essential for cold adaptation in *A. thaliana* (Dyson et al., 2016). Diamide treatment was recently shown to decrease the fumarate hydratase and L-malate hydratase activity of both recombinant FUM1 and FUM2 from *A. thaliana* (Zubimendi et al., 2018). Addition of DTT allowed the two isoforms to recover their enzymatic activity. Diamide treatment also induced the formation of high molecular weight aggregates. These aggregates could be reduced by incubation with *A. thaliana* leaf extracts supplemented with NADPH, suggesting reduction of aggregates via a TRX and NADPH-dependent TRX reductase mechanism. LC-MS/MS analysis also demonstrated the occurrence of a Cys339–Cys339 disulfide bond on FUM2.

NAD-dependent Malate dehydrogenase (NAD-MDH) catalyzes the reversible oxidation of malate to oxaloacetate while using NAD^+^ to form NADH. NAD-MDH isoforms are localized in the cytosol, mitochondria, peroxisomes, and chloroplasts. Chloroplastic NADP-dependent MDH has been shown to be redox regulated via disulfide bonds by the chloroplastic ferredoxin-TRX system on both its N- and C-terminal extensions (MiginiacMaslow et al., 1997; Carr et al., 1999; Lemaire et al., 2005). NAD-MDHs do not have these redox regulated extensions in their sequences. Nevertheless, mitochondrial MDH (mMDH) was shown to form disulfide bonds upon oxidative treatment with 50 μM CuCl_2_ (Yoshida and Hisabori, 2016). However, kinetics parameters of oxidized mMDH were not significantly affected compared to the reduced form. Moreover, mitochondrial TRX*o1* was not able to reduce oxidized mMDH. It was then suggested that mMDH is probably not a redox-regulated enzyme, and that variations of the ATP/ADP ratio were a more effective mechanism for regulation of the enzyme activity (Yoshida and Hisabori, 2016).

While mMDH principal function is to participate in the TCA cycle, cytosolic MDH (cMDH) participates in the malate valve that allows transfer of reducing equivalents between subcellular compartments (Scheibe, 2004). Recombinant cMDH could be oxidized using 50 μM CuCl_2_ while forming intermolecular disulfide bond in the dimer structure (Hara et al., 2006). Oxidized cMDH could be reduced and reactivated by DTT and TRX*h1*. Site-directed mutagenesis showed that mutation of Cys330 to Ser prevented the enzyme from being inactivated by CuCl_2_ and from forming intermolecular disulfide bonds while other mutants behaved similarly as the wild type (Hara et al., 2006). Recently, recombinant cMDH from *A. thaliana* was shown to be inhibited by H_2_O_2_. Following this treatment, cMDH could be reactivated by five different cytosolic TRX isoforms (TRX*h1-5*) (Huang et al., 2018). The inhibitive effect of H_2_O_2_ on MDH activity in purified cytosolic fractions was not observed with MDH from purified mitochondrial fractions, consistently with previous results (Yoshida and Hisabori, 2016). *In vivo* MDH activity was reduced in mutant plants deficient in the activity of the H_2_O_2_ scavenging enzyme catalase (*cat2*) and in TRX reductase deficient double mutants (*ntra/ntrb*), which display lower TRX*h* activity (Huang et al., 2018). This work also confirmed the presence of a Cys330–Cys330 intramolecular disulfide bond in the oxidized protein structure. Cys79 was found to be overoxidized to sulfenic acid. However, this modification did not seem to affect cMDH activity. The authors proposed a redox mechanism where Cys330 of one subunit makes a disulfide bond with the Cys330 from another dimer leading to an unstable tetramer. In this context, the original dimer structure would dissociate, leading to newly formed dimers linked by a Cys330–Cys330 disulfide bond (Huang et al., 2018). Interestingly, literature data have shown that overexpression of MDH in different plant organisms lead to a higher tolerance to different stress via an increase in malate synthesis (Tesfaye et al., 2001; Chen et al., 2015; Wang et al., 2016).

### Possible Indirect Impact of Oxidative Stress Through Effects on Enzyme Regulatory Phosphorylation

Protein redox modifications discussed above are a direct consequence of oxidative stress on protein properties. However, oxidative stress can also impact enzyme properties indirectly. For example, *S*-glutathionylation of TRX*f* in chloroplast could alter its oxido-reductase activity toward targets, affecting them indirectly (Michelet et al., 2005). The SnRK1 is an essential regulator of plant metabolism by direct phosphorylation of a number of enzymes involved in carbohydrate metabolism and nitrogen assimilation (Piattoni et al., 2017; Wurzinger et al., 2018). Recently, it was determined that *A. thaliana* SnRK1 kinase activity is sensitive to inhibition by H_2_O_2_. Mutation of Cys200 prevented inhibition of the SnRK1 kinase activity, suggesting this residue as a target site for redox modification (Wurzinger et al., 2017). These results suggest that redox regulation of SnRK1 could then indirectly affect its phosphorylation targets.

Another impact of oxidative stress on protein phosphorylation could involve oxidation of methionine residues to methionine sulfoxide. Phosphorylation of nitrate reductase Ser534 in *A. thaliana* was shown to be dependent on the redox state of Met538. The oxidized form of this residue prevented enzyme phosphorylation (Hardin et al., 2009). These results suggest that oxidative stress can not only induce redox modification in enzymes structure but can also indirectly interfere with other types of modifications such as phosphorylation. A cross-species survey suggests that nitrate reductase is probably not a unique occurrence for this proposed mode of regulation in plants (Rao et al., 2013).

## Formation of Protein Complexes

Besides the direct modification of metabolic enzymes, the formation of metabolic complexes has also been suggested as a way to control carbon flux in plant cells. It was first found that glycolytic enzymes were able to associate at the outer surface of mitochondria in *A. thaliana.* LC-MS/MS analysis showed the presence of hexokinase, FBA, cTPI, GapC1 and GapC2, phosphoglycerate mutase, ENO and pyruvate kinase in purified mitochondrial fractions. Moreover, the activity of all glycolytic enzymes was detected in purified mitochondria. Additional evidence came from the use of YFP-fusion proteins to show colocalization of FBA and ENO with mitochondria (Giegé et al., 2003). Later, association of glycolytic enzyme with mitochondria was shown to be dependent on respiration rate in *A. thaliana* and *S. tuberosum.* Indeed, stimulation of respiration increased glycolytic enzymes activity associated with purified mitochondria samples while inhibition of respiration had the opposite effect. Addition of nitrite to stimulate carbon flux through the PPP also increased association of glycolytic enzymes with mitochondria (Graham et al., 2007). This latter experiment suggests that during conditions where NADPH is required, (e.g., for nitrite assimilation or during oxidative stress), association of glycolytic enzymes with mitochondria could be stimulated in order to favor the PPP. There is also evidence of substrate channeling implying the presence of a functional glycolytic metabolon associated with mitochondria (Graham et al., 2007). Pull-down experiments suggested interaction of FBA with GapC but also with a VDAC located in the outer membrane of the mitochondria. These latter results suggest a mechanism where glycolytic enzyme could form a metabolon anchored at the outer membrane of mitochondria (Graham et al., 2007). FBA6 and GapC were initially found to preferentially interact with VDAC3 by far western blotting under oxidizing conditions *in vitro*, while addition of DTT prevented the interaction (Wojtera-Kwiczor et al., 2013). This study also showed interaction of FBA6 and GapC with actin under oxidizing conditions, supporting an earlier report in which FBA was proposed to interact with cellular structures (van der Linde et al., 2011). Interaction of FBA with actin has also been shown to decrease its activity (Garagounis et al., 2017). Recently, the conditions under which glycolytic enzymes bind to the mitochondria were revisited in *A. thaliana*. Colocalization of GapC with mitochondria was shown to be increased under reducing conditions compared to oxidative conditions (Schneider et al., 2018). The authors also used VDAC3 incorporated into a lipid bilayer to show an increase in binding properties with GapC under reducing conditions. These results differed from those previously obtained by far western blotting and could it be rationalized that the latter method does not represent native conditions for protein interaction. The authors proposed a mechanism in which glycolytic enzymes would be associated with mitochondria under normal conditions to provide pyruvate for respiration. Under stress, oxidized GapC would be relocated to the nucleus where it would serve to enhance defense-response genes. This mechanism would also lead to increased flux through PPPs producing NADPH as reducing power. Plant-specific non-phosphorylating GAPDH (GapN) was also proposed as an alternative to produce NADPH by the glycolytic pathway under oxidative stress condition (Schneider et al., 2018).

Formation of large protein complexes such as glycolytic enzymes anchored at the outer membrane of mitochondria appears to occur during stress and could be playing essential functions. A well accepted explanation for the formation of these complexes is to reduce the availability of intermediates in competing pathways. Metabolite channeling could help redirect the carbon flux through PPP while reducing some biosynthetic pathways. However, the impact of these complexes on metabolites and C flux regulation remains unclear and further work will be needed to elucidate the importance of this mechanism in response to oxidative conditions.

## Changes in Metabolite Pools and Metabolic Fluxes

Changes in cellular metabolites during *A. thaliana* response to oxidative stress induced by non-lethal menadione treatment of cell cultures has revealed an adaptative strategy based on the modulation of carbon fluxes through primary metabolism. Menadione is a redox-active quinone that provokes oxidative stress though the production of superoxide (Sweetlove et al., 2002). Menadione treatment was shown to increase the abundance of 6-phosphogluconate and ribose-5-phosphate suggesting a redirection of carbon flux through PPP (Baxter et al., 2007). These changes were highly dynamic as the abundance of these metabolites increased in the first hours of the treatment and then returned to the control levels. A rapid accumulation of 3-PGA was also consistent with inhibition of downstream fluxes. Accordingly, malate abundance decreased in these conditions. Kinetic analysis of ^13^C-labeled compounds following labeling with ^13^C-Glc suggested a reduction of C flux through the TCA cycle because of decreased labeling for citrate, isocitrate, 2-oxoglutarate, succinate, fumarate and malate. The metabolomic response of *A. thaliana* to oxidative stress was further studied using roots similarly treated with menadione (Lehmann et al., 2009, 2012). As previously observed, a rapid decrease of TCA cycle metabolites and an increase in PPP intermediates such as ribose 5-P and ribulose 5-P was measured after the occurrence of stress (Lehmann et al., 2009, 2012). Citrate abundance decreased at 0.5 h after menadione treatment but increased after 2 h compared to the control. In these conditions, ACO activity was also shown to be inhibited (Lehmann et al., 2012). ACO inhibition, as well as accumulation of citrate was also observed in *A. thaliana* seedlings during oxidative stress (Obata et al., 2011). As mentioned above, high citrate levels have been proposed as a response mechanism to control ROS concentrations in plant cells (Gupta et al., 2012). In contrast to results obtained in cell culture, root treated with menadione showed a decrease in levels of glucose-6-phosphate and fructose-6-phosphate. Similar results showing a decrease in glucose-6-phosphate, fructose-6-phosphate fumarate and malate was observed in seedlings exposed to menadione (Obata et al., 2011). In roots, pyruvate and pyruvate-derived amino acids were shown to accumulate under oxidative conditions (Lehmann et al., 2009, 2012). A ∼5-fold increase in pyruvate as well as an increase in citrate levels was also observed in stressed seedlings (Obata et al., 2011). This build-up of pyruvate probably reflects the impact of TCA cycle inhibition on the lower part of glycolysis. It was also shown that metabolites from glycolysis could recover much faster from oxidative stress than metabolites from TCA cycle. The authors suggested that glycolytic enzyme inhibition was limited while inhibition of TCA cycle enzymes such as ACO was stronger and irreversible (Lehmann et al., 2012). Interestingly, addition of DTT to *A. thaliana* leaves, caused opposite metabolic effect to observations made during oxidative stress. In this case, metabolites in the first part of the TCA cycle (aconitate, isocitrate, 2-oxoglutarate) increased as well as succinate, fumarate and malate (Kolbe et al., 2006). Pyruvate levels in DTT-treated tissues were also shown to decrease in contrast to observations made on menadione-stressed roots. Taken together, these results suggest a tight redox regulation on carbon flux through glycolysis and TCA cycle.

Overexpression of the cell death suppression factor, BI-1, is known to induce tolerance to different stresses (Kawai-Yamada et al., 2004). In rice, overexpression of BI-1 was shown to act by enhancing tolerance of cell culture to cell death induced by oxidative stress (Ishikawa et al., 2009, 2013). The authors used overexpression of BI-1 to better understand how plants cope with oxidative stress. During the first hours of menadione treatment, a peak of H_2_O_2_ production was observed in cell cultures. As previously observed in *A. thaliana*, the authors found that menadione treatment in rice led to an increase in PPP intermediates and in pyruvate while most metabolites from the TCA cycle were decreased. Several differences in metabolites were found between BI-1 overexpressors and control cells after 24 h of stress, including higher concentrations of GSH and GSSG, sugar phosphates, NADH, NADPH and nucleoside triphosphates. The difference was particularly remarkable for NADPH, which was seven times higher in overexpressors as compared to the control cells. These results support the hypothesis that oxidative stress leads to a metabolic shift allowing more production of NADPH thus enhancing plant tolerance to stress. The importance of NADPH generating dehydrogenases in plant adaptation to nitro-oxidative stresses has been previously emphasized (Corpas and Barroso, 2014). Not only does NADPH participate in the support of biosynthetic pathways, but it is also as mentioned above, a key player in the cellular detoxification of ROS due to its role as reductant in the ASC/GSH cycle (Noctor et al., 2017).

Studies on photo-oxidative stress and exposure to UV-B which are known to induce oxidative stress has shown common metabolic responses with observations made on menadione-treated cells (Kusano et al., 2011). These results suggest that different stresses probably have an early-response consisting of oxidative regulation of primary carbon metabolism. Indeed, several biotic and abiotic stresses are known to cause an increase in ROS concentrations (Apel and Hirt, 2004).

## Summary

In conclusion, post-translational redox modifications of enzymes from glycolysis and the TCA cycle could play critical roles during the early response of plants to oxidative stress. Inhibition of FBA, cTPI and GapC (Figure 2) would favor carbon flux through PPP in order to produce NADPH necessary to cope with an increase in ROS levels. In support of this, flux measurements and metabolic modeling made using transgenic roots with low cTPI activity (mimicking the effects of *S*-glutathionylation of the enzyme) showed increased flux through the PPP (Dorion et al., 2012; Valancin et al., 2013). Low cTPI and GAPDH activity has also been correlated with higher resistance to oxidative stress as well as increased flux through PPP in non-plant systems (e.g., *S. cerevisiae* and *C. elegans*) (Ralser et al., 2007).

Available data from multiple studies indicate that the TCA cycle is deeply affected by oxidative stress. The primary cause seems to be an inhibition of several TCA cycle enzymes (Figure 2), leading to perturbation in C flux. Redox dependent inhibition of TCA cycle enzymes such as ACO seems to cause an increase in citrate concentrations Accumulation of citrate is thought to play several roles during stress including induction of alternative oxidase to reduce ROS production. Inhibition of the TCA cycle could be beneficial during stress by reducing ROS production due to respiration.

## Author Contributions

SD drafted the article and produced the artwork. JR revised the manuscript. JR and SD both approved the final version of the manuscript.

## Conflict of Interest Statement

The authors declare that the research was conducted in the absence of any commercial or financial relationships that could be construed as a potential conflict of interest.

## Abbreviations

ACO: aconitase
ADH: alcohol dehydrogenase
ASC: ascorbate
BI-1: Bax inhibitor-1
BioGEE: biotinylated glutathione ethyl ester
BioGSSG: biotinylated oxidized glutathione
BSO: buthionine-(S,R)-sulfoximine
Cd: cadmium
cTPI: cytosolic triosephosphate isomerase
DHS: dihydrosphingosine
DTT: dithiothréitol
ENO: enolase
ESI-MS: electrospray ionization mass spectrometry
ETC: electron transport chains
FBA: fructose-bisphosphate aldolase
FUM: fumarase
GapC1/C2: cytosolic GAPDH isoforms
GAPDH: GAP dehydrogenase
GRX: glutaredoxin
GRXC1: glutaredoxin C1
GRXC2: glutaredoxin C2
GSH: reduced glutathione
GSNO: *S*-nitrosoglutathione
GSSG: oxidized glutathione
H_2_O_2_: hydrogen peroxide
H_2_S: hydrogen sulfide
IDH: isocitrate dehydrogenase
KO: knock-out
LC-MS/MS: liquid chromatography-tandem mass spectrometry
MDH: malate dehydrogenase
NaHS: sodium hydrosulfide
NO: nitric oxide
PLDδ: phospholipase D
PPP: pentose phosphate pathway
PTM: post-translational modification
RNS: reactive nitrogen species
ROS: reactive oxygen species
SnRK1: sucrose non-fermenting 1-related protein kinase
TCA: tricarboxylic acid
TRX: thioredoxin
VDAC: voltage-dependent anion channel
